# Record of Meteorological Conditions at Buffalo, for Oct., 1855

**Published:** 1855-12

**Authors:** Sanford B. Hunt


					﻿ART. VI. — Record of Meteorological Conditions at Buffalo, for Oct.,
1855. By Sanford B. Hunt, M. D.
OQ
M
s
M
PS
£
o
______________________________________________________________________ro_____________________________
•aiTUB-iadoiax I
io aStrei asaiB.unt— x c> — co no yy x x ei x —lymco t-c ct a x ct t- t-	—
’_______ J ______________Ci	—• —. —I —	—■	—	—<	Ci	Ci	Ci	—<	__________________________—
A'n IX — X	CT CT X —	CT.	-?	C) L.-; ~ l'.II	ci	-f MO ifl	O ® f CO	X	fo
mrrmr,	H1	- O t- t-O M	Ifiint-tOfftCOOCT	0 0	X	Ci
-piainR	ttBajy-,	co co op x x oo	x	t^ x co t- x t-	x co	t- x x	x	co t- t—	o- _[x
a	co	-O'	o	f . irt	xt-yyt— —. —	xx	, h ®	t-	k co-^j	ci	co
5	A\9(j	usaft	t-	—i ci t— x —■ no	co	oddy y d-r	t-d	x x‘ x	-y	—i x ci	cd	-4
2	 _________ X	LO	LO	Irt C-j<-f	XXXXyyXXxji	•?. Tf CO	CO	CO CT M	co_______yy
•aanjB	I x . coco	t— .	coco. t- t— t— co	t— t— t-	. t— co co	co
-•iadtnaj, UBdjt o	-y	a	ci ci t4 —	a	t—’ co co ci yy	co	yy no	cd co ci	o nd x ci	no	co
_______________co	lo	lo	a m -ji ■»	i.o	y x x y y	y	y i.n	x a y	y ct « y	yy_______yy
,	ct	in	v	o >n <?t -	c;	x ct — Ci ct	c - Ci	<ct —	cti-ctx	x	Fct
•AiiptuinH “f	ci	ct	ct ct x ct	i-	oo-cn-yiCCTco	—U'-^ccncc	x	x
K -r-	X CT> CT> CO X X CT) X no O CO X CO X X X !'t'O O X X i' i—	jx
=	.lnt0 T «-in	00	iN	Cl CJ	- h	ns of h co 00 c. co	X x	Cl	— X . 1—
c	i.d' Ugo	no x’ x —	ct x	ci	xxt—ocindt—ci	c >n	a	4 oc ct ®	'	ci
•	tr	x	'y lo no	to	m	cixxyyxxyyx	ct x	x	x C7 x x	ct	ct
6	£ >	i "	’ ’	—	---------------- —	- ’	—2
Cu Mn,‘ max .-J d d d a a h a o x o’ x ct ci CT x x co x t— x* x no oo	t4
____‘co Ty co r?< m to	10 co co co	-•<_________________________
.35 S'cfL’®	. £	• •	....>*
5	___________—1 —1	' ci x c x o x —< — ci ct ct o o x x ct —i —i nd nd — cd____________________________
g spOO[3JO J.aiy|X O CO CO O O CT) CT) — CT~CTCi X CT CT ct’ct CTl I- CT CT X CT' CT CT CT	|
m	{4	£2 f“ CT ci co x x ct cs^BT-r^yFordsc* qq — oo eft— —i —< oeft- c? t—CT4_ct ict
x4	• .'unTirmr, iSi£'52'^QO<^c“'^t^x—onxxciCTcoxoxodx—<t—cnxCTXX—t-
—	. Ajiptuinji x x x a? i— xt— t- t— t-t— noxxxnonoxxt— xt— xcot— nononononot— t—
p	| —— ----------------------------------------------------------------------—
c -jujot Alan PQ °°. x x x no . a. t—_ — x <CT CT x c? x co co co co . no Ci co —; x .co co—J co
g t.d. u|x x cd x cd x x cd ei >d ct ct x x on x' x ci x’ —i x't— ct x t- —i x ci x ct ci cd
•	u	IXXXXCTCTCTXXXCTCIXXCTXCTXXXXXXXCIXCTXXXCT CT
S £j ”	g*
. - -airt) dtuaj, _*•	_*•	—; —;	Ld —' ct — x' cd x’ x' co —' co' t—' o' x t-' ci x ty cd cd co' x
ct ________I no no x no no ax ct co no xct xct x CT CT x to to a ct ct x x_ct ct ct x x ko x
8-ici -d	u- •	. .^ .& .
= §§°2	ccai^^ca tzi
............. ........................... ..............................................
_______	- - y CT —' CO 1.0 Clj-“_o -y Cl -ty — co — — CO CO — —I 07 07 CO — -y KO CO Cl —' —■_
gpno[3 jo j.tny |“r5OSa02S2o<?,2So2 2 ° ° ~' ° °	2 2 2 2 o xt— ct. — o x
c CTi.n-y ac ~. o a cot- t— xxt— — co — t— co—i — t~t~x — t— —ixr-’ no"
4 AtSP'OAOH coxcocoxctococt. xcoxxxnot-nocoxt-xcot-t—xxnot—xconox x
©
-----------------— --------------------------------—_—.— ............. ■ - — ■ ■. ■   
•	2	H
=	-intoj Man	’“'cd'-'	t-	CO	07 *y KO cy	CO	CO	CT1 CO to KO	CO	t-	XX CT —; CO	CO	-	co .KO	.CO	.	I—
•	o	4	.ud M i(j	ko -y	-y	—'	-y	t4 t4 o' cd	-y	ci	cd ci x co	ci	x’	co' no' r- co ko	ko	—	ko t^—y ko	co	cd	co	co
«d	tl	a a	i.n	*y	a	a m y o	o	—	m a ci c	~	Ji	y y — k. c*.	co	ct	ci ci x x	x	x	x	x
. ainj.duiax t4 qq —J yi x' ct ct x' x t—’ t4 ct x ct id cd oi ct' >d _y ko cd cd t4 ci ct ci —' to'xcd id
t— ________a a a y a a y y y y y co ct y y y ct in m x ko ^y -y x x x -y ~y x -y x dy
g^S.
S o ®S ^^'<72 02	02 «2 CO 02	X 02 02	02	«2 02	02 02 02 I
Q ?	.	..	..	.................
_________o m 4 o oi co ci co 07 o co_co_® — co co coj>] ^2^	!
spnoo jo4.tuv|ooooooooaoo<^ooooQooooouO'^ooociooooo|
I	r“<
C •iim.ircT IO -iitI	r-i r-<	.	. CO 07 (77 07 00	.	.
“loociojoi^aidoooodooooooooooooocjoooo a>
~	________Ico C7	09 <77 (7? 09 CO CO CO	CO CO CO CO CO CO	CO CO CO CO 07 CO CO CO CO CO C-? (7? CO CO	CO 09
O	O	C r-H	0 1.0 10	CJOiOtO
q	vzx ™	CO CO	o	CO	09	co
UPHIJO*UI M	CO co to	090909	09	09	r-jr-t	09
..............................   9	_______________________9	_______________________CO
•fmnrl —'09CO'^tQCOt^OOOOr-HG9CO^fUOCO^OOOO—'09COx^iO-Ot^90C^O^|
__________ 4111______________—«	—« —> —-------' 0>> 09 09 09 09 09 C? 09 09 77 C" CO I
				

